# GuideLiner Balloon Assisted Tracking for deep cathether intubation to address challenging distal coronary lesions

**DOI:** 10.21542/gcsp.2023.7

**Published:** 2023-01-30

**Authors:** John M. Thiele, Mohammad Mathbout, Valerian Fernandes

**Affiliations:** 1 Department of Internal Medicine, Medical University of South Carolina, Charleston, South Carolina, USA; 2 Division of Cardiovascular Medicine, Department of Medicine, Medical University of South Carolina, Charleston, South Carolina, USA

## Abstract

As the medical treatment and survivability of coronary artery disease improve, patients requiring catheter-based coronary intervention present with increasingly challenging coronary anatomy. Navigating complicated coronary anatomy requires a diverse armamentarium of techniques to reach distal target lesions. Here, we discuss a case in which we used GuideLiner Balloon Assisted Tracking, a technique formerly used to achieve difficult radial access, to facilitate delivery of a drug-eluting stent to a challenging coronary target.

## Introduction

With aging populations and persistent native coronary artery disease progression, percutaneous coronary intervention (PCI) is often complicated by difficult device delivery to distal hemodynamically significant lesions. Factors affecting device delivery include tortuous vascular anatomy, obstructive proximal coronary lesions, and prior interventional devices that obstruct access. Advances in the technology implemented in complicated percutaneous interventions have addressed many obstacles encountered in difficult cases. Rotational atherectomy and shockwave lithotripsy have offered new options for plaque modification and lesion preparation for obstructions that may not resolve with traditional balloon angioplasty ^1,2^. Recent studies have touted the advantages of increased catheter support via deep intubation of guide catheters in cases of difficult stent deployment^3^.

The GuideLiner catheter (Vascular Solutions Inc., Minneapolis, MN, USA) is a “mother and child” coaxial system with a larger diameter guide catheter intended to extend the reach of traditional guide catheters for increased support to facilitate the delivery of interventional devices and stents. Deep intubation of larger guide catheters has been shown to increase backup support and simplify stent deployment to complex distal lesions^4–6^.

**Figure 1.**
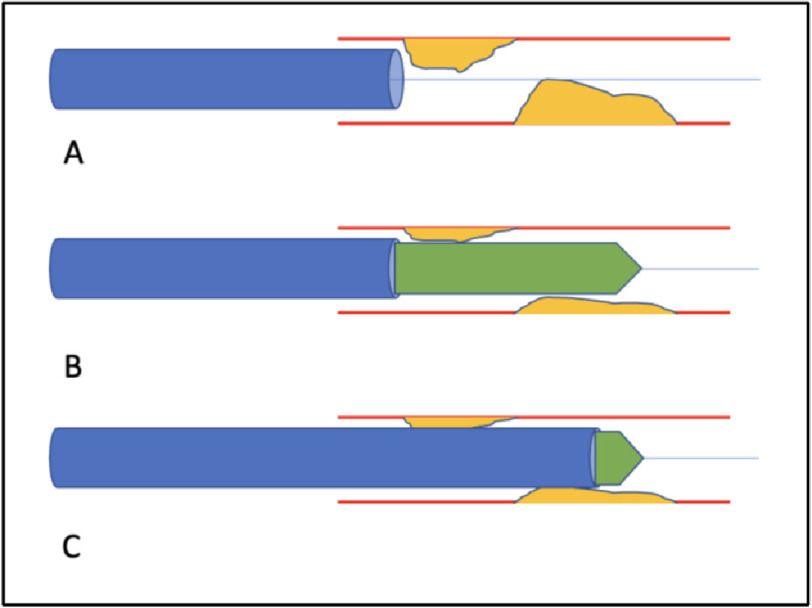
GuideLiner Balloon Assisted Tracking. (A) GuideLiner with limited accessibility to distal coronary vessel obscured by proximal obstructing lesions. (B) Balloon deployment with 1/2 remaining within Guideliner, inflated to nominal pressure. (C) Advancement of GuideLiner over balloon to bypass proximal lesions for deep intubation.

Advancement of large-diameter guide catheters can prove challenging when facing obstructive proximal coronary lesions, coronary tortuosity, or jailed target-branch ostia. One technique to aid catheter passage has been dubbed “GuideLiner Balloon Assisted Tracking” (GBAT). This is performed by advancement of a balloon halfway through the tip of the GuideLiner and dilation of the stenotic coronary artery, with the balloon inflated to nominal pressure. Thereafter, the GuideLiner is advanced over the balloon to the target lesion and the balloon is deflated for deployment of interventional devices or stents (Figure 1)^7^.

We report on a 72-year-old male with recent left main coronary artery and left anterior descending artery (LM/LAD) stenting with severe diffuse left circumflex artery (LCx) disease, terminating in a severe ostial obtuse marginal artery (OM) lesion. Device access was limited by a tortuous LCx, including several acute deflections, a jailed LCx ostium, and diffuse obstructive lesions proximal to the distal ostial OM lesion.

Dilation of the strut window at the ostium of the LCx was performed, and a GuideLiner catheter was advanced deep into the LCx using GBAT with successful stenting of the ostial OM and diffusely diseased LCx.

### Case

A 72-year-old male with past medical history including hypertension, hyperlipidemia, hepatitis B, prior tobacco and alcohol abuse, and coronary artery disease (CAD). He had undergone three-vessel coronary artery bypass grafting (CABG) (LIMA-LAD, SVG-OM, SVG-distal RCA) 11 years prior, with atherectomy and stenting of LM-LAD lesion 2 months prior. He presented for continued dyspnea on exertion and stable angina, despite successful PCI to LM/LAD lesion two months ago.

**Figure 2.**
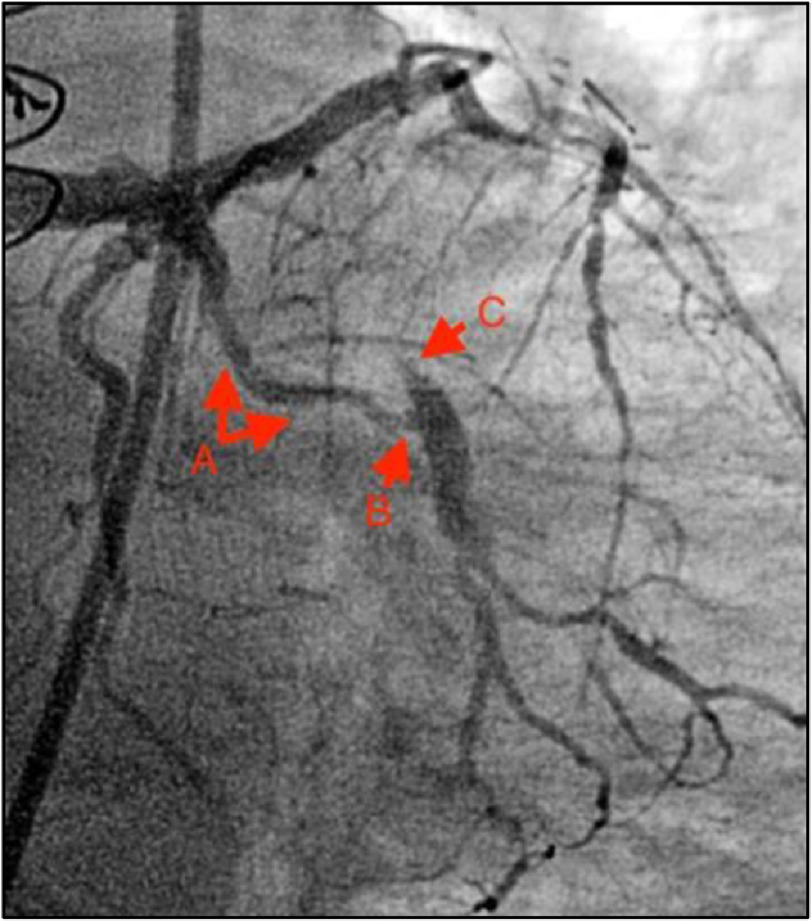
Preprocedural coronary angiography of LMCA depicting (A) diffuse severe disease of LCx, (B) severe lesion at the ostium of OM1, and (C) SVG-OM1 graft known to be occluded.

Coronary angiography demonstrated patency of the recently placed LM/LAD stents, a known occluded saphenous venous graft to the obtuse marginal artery (SVG-OM), and severe diffuse LCx disease with focal severe stenosis at the ostium of the large-caliber OM (Figure 2). A decision was made to proceed with PCI.

**Figure 3.**
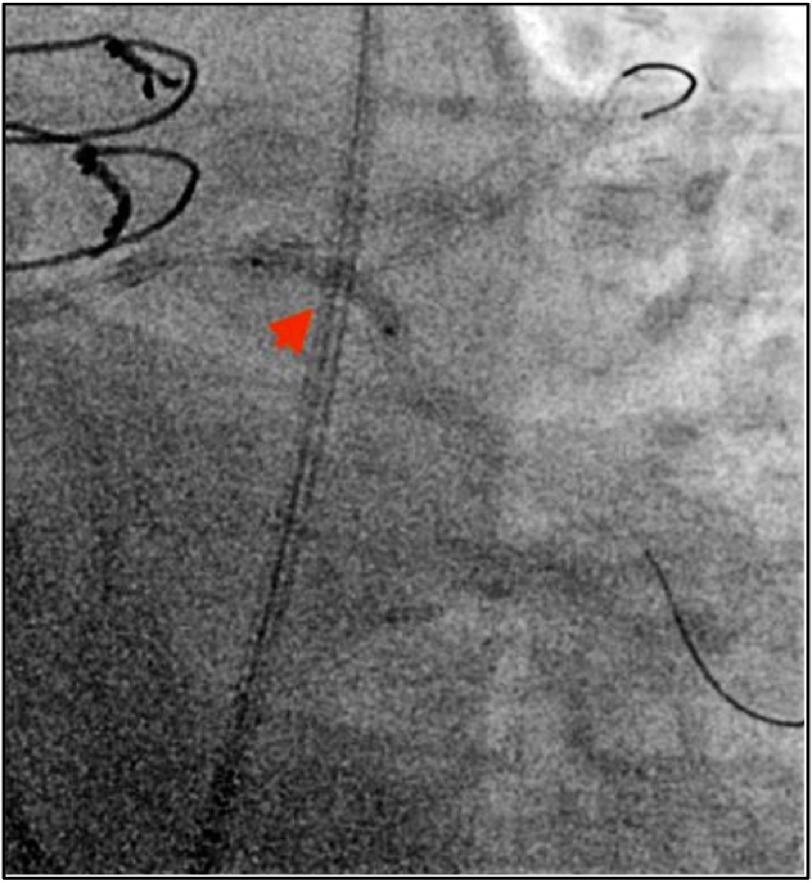
Strut window dilation of prior LAD stent via 1.5 mm and 2.5 mm Sapphire balloons (Orbus Neisch, Netherland).

The patient was scheduled for PCI for the LCX and ostial OM1 lesions. A 7Fr EBU guiding catheter (Medtronic, Dublin, Ireland) was used to engage the LM. Two Runthrough wires (Terumo, Tokyo, Japan) were used to sequentially engage the LAD and LCx/OM. First, the struts of the existing stent in the LM were dilated with 1.5 and 2.5 mm Sapphire balloons (OrbusNeich, Hong-Kong, China) to optimize passage of a GuideLiner (Vascular Solutions Inc., Minneapolis, MN, USA) into the LCx and to the OM lesion (Figure 3).

**Figure 4.**
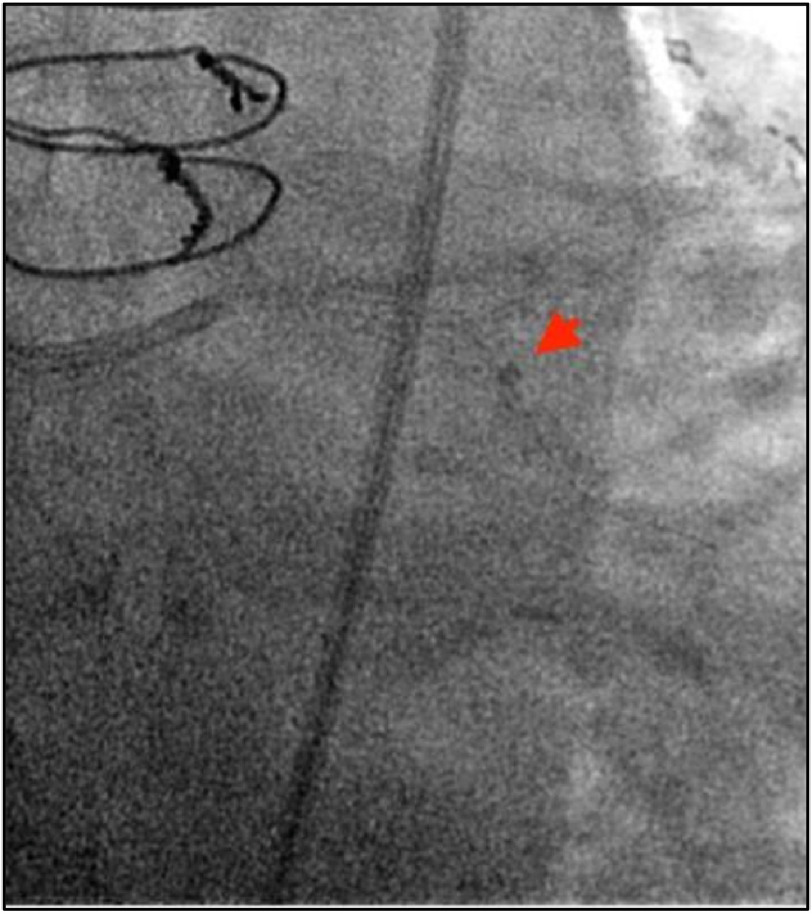
GuideLiner Assisted Balloon Tracking technique used to advance GuideLiner catheter along severely diseased and tortuous LCX. Pictured advancing past mid-LCx.

Passage through the LCx was complicated by obstructive proximal LCx coronary lesions and significant tortuosity of the native LCx. GuideLiner Balloon Assisted Tracking was used to advance the GuideLiner through the LCx to the ostial OM lesion (Figure 4).

**Figure 5.**
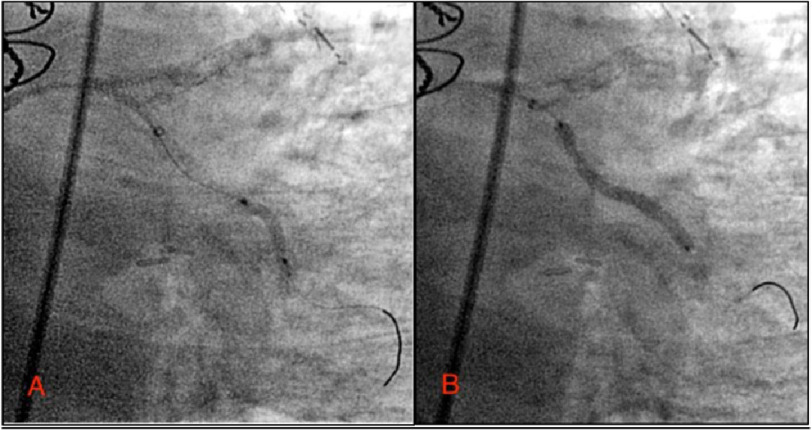
Pre-dilation of distal OM lesion (A) and 34 mm stent placement to LCX/OM performed via deployment and retraction of the GuideLiner catheter (B).

**Figure 6.**
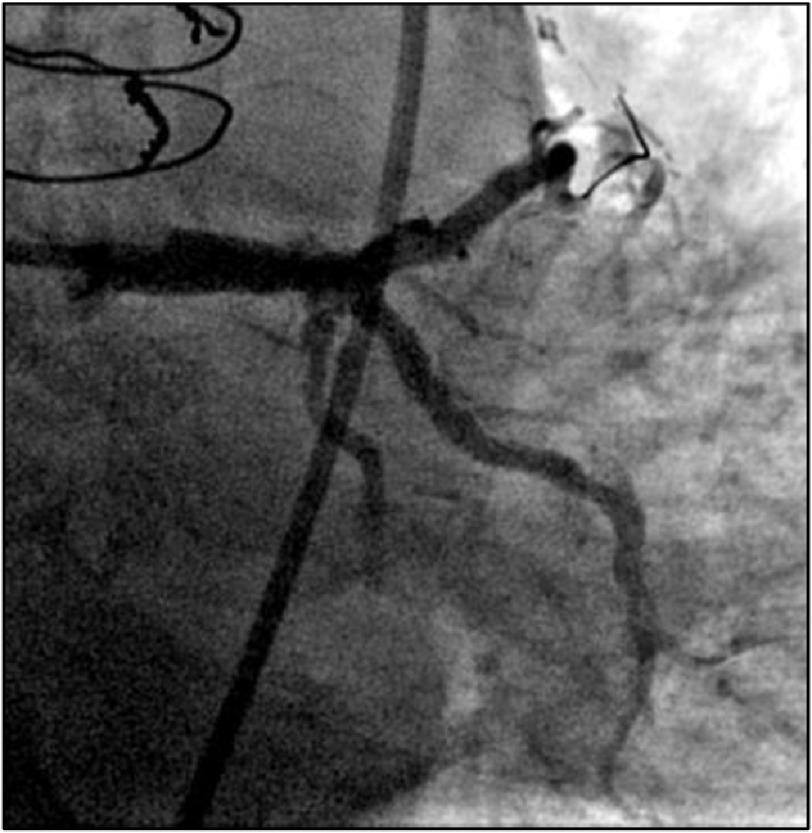
Revascularized LCx/OM demonstrating minimalresidual stenosis after deployment of drug eluting stent.

The severely calcified OM lesion was pre-dilated with 2.5 mm Emerge balloon (Boston Scientific, Marlborough, MA, USA), and stented with 2.5 ×34 mm Resolute Onyx (Medtronic, Dublin, Ireland) (Figure 4). The stent was post-dilated with a 3.25 mm non-compliant Emerge balloon (Boston Scientific, Marlborough, MA, USA) at high pressure with resultant TIMI-3 flow and minimal residual stenosis on angiography (Figure 5, Figure 6).

## Discussion

In this case, we present an instance of the successful utilization of jailed side-branch strut window dilation and GBAT with deep GuideLiner intubation to reach distal coronary lesions. The anatomy of our patient prior to the intervention demonstrated accumulation of coronary lesions in severe, chronic coronary artery disease. With the aging of patient populations, complex coronary anatomy has become increasingly prevalent. However, as technologies and skills in the realm of percutaneous intervention continue to progress, more complex lesions can be addressed with PCI, avoiding surgical coronary bypass grafting in patients at high risk of perioperative adverse events associated with open procedures.

GBAT is derived from balloon-assisted tracking (BAT), which has been reported as a method of establishing or maintaining transradial access in patients with tortuous vascularity or refractory radial artery spasms. In instances of complex radial artery access, GBAT greatly reduced the necessity of conversion to a transfemoral approach and demonstrated no delay in revascularization when compared to conversion to a transfemoral approach^8^. These promising data in using BAT in arterial approaches have led to the adoption of this technique in coronary device delivery in patients with complex coronary anatomy. Deep intubation with guide catheters provides enhanced device support and can increase the success rate of PCI^9,10^. However, deep guide catheter intubation is not without risks. Examination of complications of GuideLiner catheters revealed that 23% of complications were attributed to difficulty advancing the catheter, complicating successful stent delivery. However, no modified delivery techniques have yet been developed. Coronary artery dissection or perforation were also noted as complications, occurring in 14% and 3% of patients, respectively^11^.

Deep intubation with guide catheters is an invaluable skill for modern interventionists. As the technology and techniques in PCI have evolved, the spectrum of complex cases that qualify for percutaneous intervention has expanded. This requires familiarity with catheter-based techniques and mechanisms to traverse the challenging coronary anatomy. Our case invokes several advanced techniques for device delivery to distal coronary lesions, including jailed-side branch strut window dilation, deep intubation of a guide catheter, and GBAT for navigation of tortuous and heavily diseased coronary arteries.

### What have we learned?

• GuideLiner balloon assisted tracking is a viable technique for traversing tortuous or diseased coronary anatomy to access challenging coronary lesions.
• Continued development of new techniques for coronary maneuverability is important to overcome challenges in delivery of percutaneous coronary interventions.
